# Dream Lucidity and the Attentional Network Task

**DOI:** 10.3389/fpsyg.2021.586808

**Published:** 2021-01-28

**Authors:** Moo-Rung Loo, Shih-kuen Cheng

**Affiliations:** Institute of Cognitive Neuroscience, National Central University, Taoyuan, Taiwan

**Keywords:** lucid dream, trait lucidity, attentional network task, conflict resolution, attention

## Abstract

This study investigated the relationship between dream lucidity, i.e., a dreamer’s insight to the ongoing dream, and attention by considering lucidity as a trait. We examined the ways in which lucidity correlates with the orienting, alerting, and conflict components of the attentional network. A total of 77 participants rated the lucidity of their dreams over 7 consecutive days with the LuCiD scale and then completed the attentional network task (ANT). A negative correlation between trait lucidity and the conflict score of the ANT was found for 49 participants whose responses were faster when an alerting signal was presented. This result suggested that, with a prerequisite that the presence of cues facilitates subsequent information processing, the greater a person’s trait lucidity, the more efficiently he or she is capable of resolving conflicts.

## Introduction

The role of attention in consciousness has been extensively examined, with relevant research focusing on whether and how attention is required for conscious perception (Dehaene et al., 2006; Koch and Tsuchiya, 2007). The majority of these studies have been conducted in awake people through methods that manipulated the phenomenal awareness of stimuli in an all-or-none manner (e.g., Lamme and Roelfsema, 2000; Dehaene et al., 2003). However, consciousness is gradual rather than dichotomous (Schooler, 2002; Overgaard et al., 2006; Kouider et al., 2010; Voss et al., 2013; Bayne et al., 2016). The gradualness of consciousness is revealed by dreams that are associated with different levels of lucidity, thereby reflecting various states of consciousness (Moss, 1986; Revonsuo, 1995). The current study considered dream lucidity as a trait and investigated the relationship between attention and consciousness from this perspective and examined the ways in which dream lucidity correlates with different aspects of attentional processing.

During non-dreaming sleep, a person is not consciously processing internal or external stimuli. However, while dreaming, the internally generated perceptual and emotional experiences of a person are usually associated with bizarre events (Hobson et al., 1987; Schredl, 2003; Scarone et al., 2007). The consciousness associated with dreams exists as a spectrum. In most cases, dreamers are deprived of logical thoughts and meaningful actions (Hobson et al., 2000; Voss et al., 2009; Mutz and Javadi, 2017). They have no control over ongoing events and lack the ability to distinguish reality from dream contents (Voss et al., 2018). Dreamers are in a primary state of consciousness (Edelman, 2003) in which the past and the future are fused into the immediate present, and they lack the ability to plan their own behaviors. However, dreams in which dreamers can decide about their behaviors and feel emotions also exist (Kahan, 1994). In extreme cases such as lucid dreams, dreamers are consciously aware that they are dreaming (LaBerge et al., 1981; Baird et al., 2019). Lucid dreamers exhibit metacognition and self-awareness—the core characteristics of secondary consciousness. Lucid dreaming has been argued to be a hybrid state of consciousness (Voss et al., 2013) that cannot be dichotomously categorized into primary or secondary consciousness.

To differentiate consciousness states associated with dreams and identify the core characteristics of lucid dreams, Voss et al. (2013) developed a self-reported questionnaire, namely the Lucidity and Consciousness in Dreams scale (LuCiD scale), to examine dreamers’ memory of their dreams. Voss et al. (2013) and Voss et al. (2018) identified eight factors to construct the LuCiD scale, among which the factors of insight, control, thought, memory, dissociation, and positive emotion differentiated lucid dreams from non-lucid dreams. The LuCiD scale has been used to examine how dream lucidity as a trait correlates with metacognition. Filevich et al. (2015) scanned the brains of participants assigned to high, medium, and low lucidity groups on the basis of the composite score obtained from the average of LuCiD scale scores over seven consecutive mornings and the subjectively reported lucid dream frequency. They reported that participants with high lucidity demonstrated higher gray volumes in the BA9/10 region, which is linked to metacognition and monitoring ability, than participants with low lucidity (Burgess et al., 2007; Fleming et al., 2012; McCurdy et al., 2013). Supported by the fMRI finding that the activation of the BA9/10 region was related to metacognition and differed between high and low dream lucidity groups, the high lucidity group was suggested to have better metacognition capacity than the low lucidity group.

Lucidity has also been linked to the function of attention. To test the hypothesis that attentional skills are required for self-awareness during lucid dreams, Blagrove et al. (2010) examined the performance of high, medium, and low frequency lucid dreamers in a color-naming Stroop task and discovered that high frequency lucid dreamers responded faster during incongruent conditions than did the other dreamers. Accordingly, high frequency lucid dreamers were considered to have better attentional skills than non-lucid dreamers. However, these findings had some limitations. First, high frequency lucid dreamers also demonstrated faster responses in the congruent condition than medium and low frequency lucid dreamers did. Therefore, whether their findings reflected a general faster response capacity or a specific attentional skill associated with lucidity was unclear. Second, that study employed the self-reported lucid dream frequency, which might not be a sensitive index of lucidity in isolation (Aviram and Soffer-Dudek, 2018). Moreover, as suggested by Voss et al. (2013), dream lucidity is determined by multiple factors that cannot possibly be indexed through the single factor of frequency. Finally, attention is a complex function with various components. Recent behavioral and functional imaging studies have suggested that at least three functional networks, namely alerting (i.e., achieving and maintaining the alert state), orienting (i.e., selecting the information from perceptual input), and conflict (or executive control, i.e., resolving the conflict information from responses), contribute to the functionality of attention (Posner and Petersen, 1990; Fan et al., 2002). Therefore, the relationship between dream lucidity and the various components of attention must be examined due to its theoretical importance.

The current study employed the attentional network task (ANT) to assess the attentional processing of alerting, orienting, and conflict (Fan et al., 2002). Participants’ dream lucidity was indexed using the combination of LuCiD scale scores and the self-reported lucid dream frequency (Filevich et al., 2015). We expected that the correlation between the composite index of lucidity and three-component scores of the ANT would explain how dream lucidity as a trait correlates with different aspects of attentional processing.

## Materials and Methods

### Participants

A total of 77 college students (aged between 20 and 35 years; 25 men and 52 women) from National Central University and National Yang-Ming University, Taiwan, participated in this experiment. All participants were right-handed native Mandarin Chinese speakers and had no history of psychiatric or neurological conditions. They reported dreaming more than three times a week and had a regular sleep pattern. Each student received 1,000 NTD (about 35 USD) for their participation.

### Materials

#### The Questionnaire of Dream Lucidity

The LuCiD scale developed by Voss et al. (2013) was translated to Mandarin Chinese and was presented electronically through a Google document. The scale comprised 28 items, and participants rated each item on a 6-point Likert scale. The 28 items were divided into eight factors: insight (awareness of one’s state), control (ability to control thoughts and actions in dreams), thought (logical thinking), memory (memory that is linked to waking life), dissociation (experiencing the dream from the perspective of a third person), realism (perceived reality), positive emotion, and negative emotion. The factors of insight, thought, memory, dissociation, control, and positive emotion were used to distinguish lucid dreams from non-lucid dreams (Voss et al., 2013).

#### The Attention Network Task

The ANT developed by Fan et al. (2002) was employed in the current study to evaluate the function of the alerting, orienting, and conflict (executive control) components of attention networks. In this task, participants were asked to judge the direction (pointing to the left or to the right) of a target arrow. Each trial started with a cross fixation displayed at the center of the screen for 100 ms. The target arrow along with four flankers was presented horizontally above or below the cross fixation 400 ms later. During the 400-ms period prior to the appearance of the target arrow, the cross fixation was either presented by itself on the screen (no cue condition), replaced by an asterisk (center cue condition), accompanied by an asterisk that was below or above it (spatial cue condition), or accompanied by two asterisks with one below and the other above it (double cue condition). Four flankers accompanied the target arrow, with two on each side of the target arrow. The flankers could be dashed lines (neutral condition), arrows pointing in the same direction as the target arrow (congruent condition), or arrows pointing in the opposite direction of the target arrow (incongruent condition). Participants provided a response on the direction of the target arrow within 1,700 ms after the onset of the arrow. Thereafter, the screen went blank until the next trial started.

#### Lucid and Non-lucid Dream Frequencies

Participants completed an online survey of the frequencies of their dreams and lucid dreams. For all dreams in general, they had to indicate the frequency of dream recall as “almost every morning,” “more than once a week,” “2–3 times per month,” “approximately once a month,” “less than once a month,” or “never.” For lucid dreams, they had to indicate the frequency of recalling lucid dreams as “more than once a week,” “approximately once a week,” “2–3 times per month,” “approximately once a month,” “approximately 2–4 times per year,” “approximately once a year,” “less than once a year,” or “never.” These two surveys and the scoring of their alternatives were adopted from Filevich et al. (2015).

### Procedure

After enrollment into the study, participants rated the lucidity of their dreams over 7 consecutive days. Each morning, they filled out the LuCiD scale within 15 min of waking up. In addition to completing the LuCiD scale, participants indicated whether they had dreamt the night before and if they remembered the dream contents. After 7 days of completing the lucid dream survey, each participant visited the laboratory to complete the ANT. The ANT comprised 3 blocks, with 96 trials in each block.

### Scoring Methods

#### Trait-Lucidity

Trait lucidity, adopted from Filevich et al. (2015), was indexed using the combination of LuCiD scores and self-reported frequency of lucid dreams. Each participant’s LuCiD score was obtained by first averaging the scores of each item across the nights participants remembered their dream contents and then aggregating the average scores of items that belonged to the factors of insight, control, thought, dissociation, and positive emotion. The lucid dream frequency reported by participants was transformed into times per month. The standardized LuCiD score and the lucid dream frequency score were then combined to produce a composite score to reflect both the quality and quantity of lucidity (Filevich et al., 2015).

#### The Alerting, Orienting, and Conflict Score

The mean response time (RT) for each cue condition and flanker condition was separately computed for each participant only on the basis of correct trials. The alerting score was obtained by subtracting the RT for the double cue condition from the RT for the no-cue condition. The orienting score was obtained by subtracting the RT for the spatial cue condition from the RT for the center cue condition. The conflict score was derived by subtracting the RT for the congruent condition from the RT for the incongruent condition (Fan et al., 2002).

### Statistical Analysis

A repeated-measures analysis of variance (ANOVA) employing the factors of cue type (no cue, center cue, double cue, and spatial cue) and flanker type (incongruent, neutral, and congruent) was conducted on the RT and accuracy of the ANT. Pearson’s correlation analysis was then separately performed between the composite scores of trait lucidity and each of the three component scores of the ANT.

## Results

### The Questionnaire of Lucidity

**FIGURE 1:**
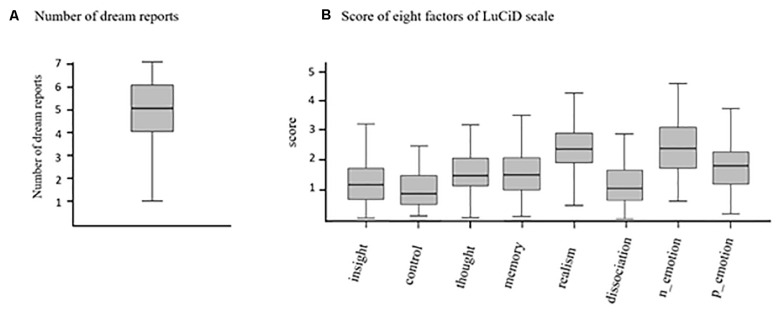
Box plots of **(A)** the number of dreams reported during the 7 days and **(B)** the scores of the eight factors of the LuCiD scale.

Figure 1A presents the number of days participants reported their dreams and completed the questionnaire of lucidity during 7 days. On average, participants reported about five dreams (mean = 4.7, *SD* = 1.6) across the 7 days. The LuCiD scale was employed to evaluate the lucidity of each dream. The scores of the eight factors were separately averaged across the dreams for each participant and then averaged across all participants (Figure 1B).

### ANT Results

Table 1 presents the accuracy rates and RTs of the correct trials for the different conditions of the ANT. ANOVA of RT revealed a significant main effect for the cue type [*F*(3, 228) = 94.334, *p* < 0.001, η*_*p*_*^2^ = 0.55]. The responses to the spatial cues were faster than the responses to the double cues [*t*(228) = 12.081, *p*_bonf_< 0.001, Cohens’d = 1.377], which were approximately as fast as the responses to the center cues [*t*(228) = 1.050, *p*_bonf_ = 1, Cohens’d = 0.12]. The responses to the center cues were faster than the responses to the no-cue condition [*t*(228) = −2.772, *p*_bonf_ = 0.042, Cohens’d = −0.316]. The main effect of the flanker type was significant [*F*(2, 152) = 694.724, *p* < 0.001, η*_*p*_*^2^ = 0.901]. The responses in the congruent condition were as fast as the responses in the neutral condition [*t*(152) = 1.383, *p*_bonf_ = 0.512, Cohens’d = 0.158], which were faster than the responses in the incongruent condition [*t*(152) = −28.206, *p*_bonf_ < 0.001, Cohens’d = −3.214]. The interaction between the cue type and flanker type was also significant [*F*(6, 456) = 2.542, *p* < 0.05, η*_*p*_*^2^ = 0.032], indicating that the magnitude of the cue type effect was different in different flanker conditions. Nevertheless, the similar cue type effect pattern (spatial cue < double cue = center cue < no cue) was observed in all three flanker conditions.

**TABLE 1:** The response times (msec) and accuracy rates for the different conditions of the attentional network task (ANT) task (SD in parenthesis).

**Flanker type**	**No cue**	**Center cue**	**Double cue**	**Spatial cue**
**Response times**				
Neutral	522 (95)	515 (92)	519 (97)	496 (101)
Congruent	529 (99)	521 (97)	517 (93)	494 (94)
Incongruent	603 (113)	600 (100)	594 (103)	566 (106)
**Accuracy rates**				
Neutral	0.96 (0.04)	0.97 (0.04)	0.97 (0.04)	0.98 (0.04)
Congruent	0.97 (0.04)	0.97 (0.04)	0.97 (0.04)	0.98 (0.03)
Incongruent	0.88 (0.08)	0.88 (0.07)	0.89 (0.08)	0.94 (0.06)

ANOVA of accuracy revealed that a significant main effect for the cue type [*F*(3, 228) = 13.017, *p* < 0.001, η*_*p*_*^2^ = 0.146]. The accuracy rate was higher in the spatial cue condition than in the double cue condition [*t*(228) = −4.757, *p*_bonf_< 0.001, Cohens’d = −0.542], and the accuracy rate in the double cue condition was statistically identical to the accuracy rate in the center cue condition [*t*(228) = −1.292, *p*_bonf_ = 1, Cohens’d = −0.147]. Finally, the accuracy rate in the center cue condition was higher than that in the no cue condition [*t*(228) = −0.009, *p*_bonf_ = 1, Cohens’d = −0.001]. The main effect of congruency was significant [*F*(2, 152) = 151.367, *p* < 0.001, η*_*p*_*^2^ = 0.666]. More errors were made in the incongruent condition than in the neutral condition [*t*(152) = −13.433, *p*_bonf_< 0.001, Cohens’d = −1.531], whereas the accuracy rate was statistically identical [*t*(152) = −0.0291, *p*_bonf_ = 1, Cohens’d = −0.033] for the neutral and congruent conditions. The interaction between the cue type and flanker type was also significant [*F*(6, 456) = 4.847, *P* < 0.001, η*_*p*_*^2^ = 0.06], reflecting that the magnitude of the cue type effect varied across flanker conditions. Nevertheless, a similar pattern of the accuracy of the cue type effect (spatial cue < double cue = center cue < no cue) was observed in all three flanker conditions.

### Correlation Between ANT and Trait Lucidity

**FIGURE 2:**
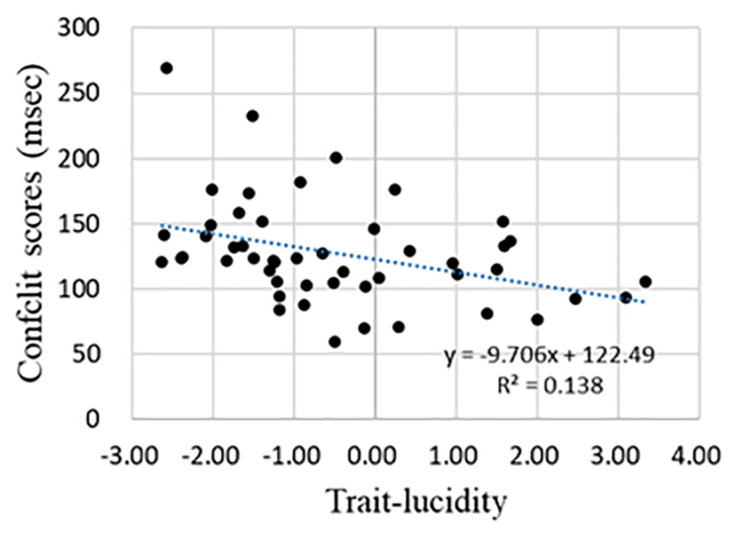
The regression curve of trait-lucidity and conflict score for participants whose alerting scores were greater than zero.

Pearson’s correlation analysis discovered that the correlation between any two of the three ANT component scores (i.e., alerting, orienting, and conflict scores, see section “The Alerting, Orienting, and Conflict Score”) was not significant. To understand the relationship between trait lucidity and attentional processing, Pearson’s correlation analysis of trait lucidity (i.e., the composite scores of lucidity and frequency of lucid dream) and each of the three component scores of the ANT was conducted. None of the three component scores were correlated with trait lucidity. However, for a subgroup of 49 participants whose alerting scores were greater than zero, a negative correlation was found between trait lucidity and the conflict score (*r* = −0.371, see Table 2). The higher the lucidity score, the smaller was the conflict score (Figure 2).

**TABLE 2:** The Pearson’s correlation coefficients between the alerting, orienting, and conflict scores of the ANT task and trait-lucidity from a subgroup of participants whose alerting scores were greater than zero (*n* = 49).

**Scores of three attentional functions (*n* = 49)**				
		**Alerting**	**Orienting**	**Conflict**
Trait-lucidity	Pearson’s r	−0.079	0.014	−0.371
	*p*-value	0.592	0.924	0.009

## Discussion

This study investigated whether and in what ways dream lucidity as a trait correlates with different aspects of attentional processing. The composite scores of the lucid dream frequency and the averaged LuCiD scale scores across 7 consecutive days were used to index trait lucidity (Filevich et al., 2015). Additionally, three component scores of the ANT (alerting, orienting, and conflict) were used to index attentional processing. The results demonstrated that trait lucidity is related to some aspects of attentional processing under certain circumstances. Specifically, for participants with positive alerting scores, a negative correlation was observed between trait lucidity and the conflict component of the ANT. This correlation, however, was not found when the alerting score was zero or negative. In other words, with a prerequisite that the presence of cues facilitates subsequent information processing, the greater a person’s trait lucidity, the more efficiently he or she is capable of resolving incongruent conditions.

Previous studies that examined the personality traits and cognitive styles of lucid dreamers found that, compared with non-lucid dreamers, lucid dreamers tend to have an internal locus of control (Blagrove and Hartnell, 2000), are more self-reflective (Purcell et al., 1986; Gruber et al., 1995), and demonstrate more independence than dependence (Gackenbach et al., 1985; Patrick and Durndell, 2004). These characteristics enable lucid dreamers to notice the inconsistencies in their dreams (Gruber et al., 1995), adopt a critical attitude toward their dream contents, sustain self-awareness, and maintain the lucid dream. The argument that the ability to identify and distinguish between reality and bizarre dream contents is essential for the commencement and maintenance of lucid dreams is supported by the discovery of a strong relationship between lucid dream frequency and the degree of field independence that reflects the ability to separate the hidden information from surrounding context (e.g., Gackenbach et al., 1985; Patrick and Durndell, 2004). Few studies have examined the relationship between cognitive styles and conflict resolution; however, an event-related potential study demonstrated that field-independent individuals processed conflict information more efficiently than field-dependent individuals did (Meng et al., 2012). The current finding of a negative correlation between trait lucidity and conflict scores further suggests that the abilities to detect inconsistencies in dream contents and maintain self-reflection in lucid dreams may be functionally related to field independence. Both of these abilities reflect the capacity to resolve conflicting information that requires the engagement of the attentional network.

The negative correlation between trait lucidity and conflict scores was found only for participants who showed a positive alerting score in the ANT. The alerting score, derived by subtracting the mean RT of the double cue condition from the mean RT of the no-cue condition, refers to the ability to achieve and maintain an alert state (Fan et al., 2002). A positive alerting score, therefore, suggests that the cue has been properly utilized to engage in and/or maintain an alert state. The relationship between alertness and the conscious report has been described in a few studies on behavior and neuropsychology. Kusnir et al. (2011) demonstrated that the detection and discrimination of near-threshold visual stimuli were improved when a warring sound was presented prior to the stimuli to enhance phasic alertness. Robertson et al. (1998) suggested that presenting a warning tone to left-neglected patients, whose attention is biased toward the right visual field due to damaged right parietal regions (Corbetta et al., 1993), enhanced the conscious detection of stimuli presented in the neglected left visual field. The importance of phasic alertness on conscious perception also gained support from a recent fMRI study. Chica et al. (2016) manipulated the factors of phasic alertness and endogenous attention with warning tones and central cues, respectively, in an experiment that required participants to detect and discriminate near-threshold visual stimuli. A midbrain-thalamic-anterior cingulate cortex (ACC) circuit linked to the anterior alerting system (Yanaka et al., 2010) was found to be involved in the interaction between phasic alertness and conscious perception. Notably, ACC has been linked to conflict resolution (Botvinick et al., 1999; MacDonald et al., 2000) and phasic alerting (Chica et al., 2016). Furthermore, Filevich et al. (2015) reported that high frequency lucid dreamers have a greater ACC volume than low frequency lucid dreamers. The negative correlation between lucidity and conflict scores was found only when the alerting score was positive, indicating that phasic alertness plays a critical role in utilizing conflict resolution capacity to maintain dream lucidity.

There are nevertheless a few caveats to be considered regarding the current findings. First, the participants were homogeneous in the sense that most of them were students aged below 35 years old. Given that young people generally perform well in the attentional network task, the failure to find correlations between trait lucidity and the orienting and alerting components of the attentional work task could be due to the insufficient individual differences in the ANT performance. It would be therefore of theoretical importance to examine whether a similar relationship between dream lucidity and attention could be revealed in other populations such as the elderly. It has been reported that young people tend to have more lucid dreams than older people (Voss et al., 2012). A relevant issue to be addressed would be whether the trait lucidity and attention performance decrease similarly as people age. It should also be noted that the current findings were correlational in nature. Future studies are needed to clarify the causal relationship between trait lucidity and the conflict component of the attentional network.

## Conclusion

The current study results suggest that lucidity as a trait is correlated with the conflict resolution component of attentional network, given that the prerequisite of the alerting system being engaged is met.

## Data Availability Statement

The raw data supporting the conclusions of this article will be made available by the authors, without undue reservation, to any qualified researcher.

## Ethics Statement

The studies involving human participants were reviewed and approved by the Research Ethic Center, National Taiwan University. The patients/participants provided their written informed consent to participate in this study.

## Author Contributions

S-kC and M-RL conceived the research idea, designed the experiment, and wrote up the manuscript. M-RL conducted the experiment and analyzed the data. Both authors contributed to the article and approved the submitted version.

## Conflict of Interest

The authors declare that the research was conducted in the absence of any commercial or financial relationships that could be construed as a potential conflict of interest.
